# A Competing-Risks Approach to the Progression, Regression and Persistence of High-Grade Cervical Dysplasia in Patients over 30 Years Old—A Prospective Study

**DOI:** 10.3390/jcm14176303

**Published:** 2025-09-06

**Authors:** Iulian-Valentin Munteanu, Demetra Socolov, Razvan Socolov, Ana-Maria Adam, Gigi Adam, Ingrid-Andrada Vasilache, Petronela Vicoveanu, Valeriu Harabor, Anamaria Harabor, Alina-Mihaela Calin

**Affiliations:** 1 Clinical and Surgical Department, Faculty of Medicine and Pharmacy, ‘Dunarea de Jos’ University, 800216 Galati, Romaniaharaboranamaria@yahoo.com (A.H.);; 2 Department of Mother and Child Care “Grigore T. Popa” University of Medicine and Pharmacy, 700115 Iasi, Romania; 3 Department of Pharmaceutical Sciences, Faculty of Medicine and Pharmacy, ‘Dunarea de Jos’ University, 800216 Galati, Romania; 4 Department of Mother and Newborn Care, Faculty of Medicine and Biological Sciences, ‘Ștefan cel Mare’ University, 720229 Suceava, Romania

**Keywords:** high-grade cervical dysplasia, advanced age, risk scores, progression, regression, persistence

## Abstract

**Background/Objectives**: In Romania, where cervical cancer incidence remains among the highest in the European Union, a risk-based management strategy could support more precise allocation of limited resources. The aim of this study was to test the prognostic utility of immediate pre-treatment and post-treatment risk predictions, derived from the American Society of Colposcopy and Cervical Pathology (ASCCP) risk-based management guidelines for the prediction of progression, regression or persistence of high-grade cervical dysplasia. **Methods**: In this prospective cohort study, we included 223 patients aged over 30 years who underwent self-referred or targeted screening with or without histologically confirmed cervical intraepithelial neoplasia (CIN) of any grade. We employed Fine and Gray’s subdistribution hazard model that evaluated the cumulative incidence function for each specific outcome, treating other outcomes as competing events. These outcomes were further stratified depending on the type of high-grade dysplasia. **Results**: The immediate post-treatment risk was significantly associated with subsequent progression of cervical dysplasia. For a cut-off of 60%, the immediate post-treatment risk was able to significantly predict the progression of both CIN2+ and CIN3+. On the other hand, the immediate pre-treatment risk > 60% was significantly associated with progression of CIN3+, but not of CIN2+. Also, the immediate pre-treatment risk was significantly associated with regression, but this observation did not persist at the >60% threshold. Both pre- and post-treatment risk > 60% were strongly associated with persistence across histologic subgroups. **Conclusions**: The ASCCP-derived immediate risk estimates, especially post-treatment risk > 60%, proved effective in predicting progression and persistence of high-grade cervical dysplasia.

## 1. Introduction

Cervical intraepithelial neoplasia (CIN) is defined as a premalignant lesion that occurs as a result of a persistent infection with human papillomavirus (HPV) [1,2]. Prompt diagnosis of high-grade CIN is imperative as it carries a significant risk of progression, and it is typically addressed with a proactive treatment approach [3,4,5].

High-grade CIN, which refers to CIN2, CIN3, CIN2/3, CIN2+ or high-grade squamous intraepithelial lesions (HSILs) according to the terminology adopted by the World Health Organization (WHO) [6,7], poses a significant public health challenge in Europe, despite ongoing initiatives aimed at decreasing its prevalence via vaccination and enhancing early detection through the establishment of screening programs. A report from the European Centre for Disease Prevention and Control in 2020 indicated that annually, between 263,227 and 503,010 cases of high-grade CIN are diagnosed within the European Union and the European Economic Area (EU/EEA) [8]. Additionally, there are over 34,000 newly diagnosed cervical cancer cases and more than 13,000 deaths reported [8].

In Romania, the incidence rate of cervical cancer is 33 cases per 100,000 people, which is approximately three times higher than the incidence rate in the European Union (12 cases per 100,000 people) [9]. This highlights the significance of immunization against HPV and the high economic burden that is associated with high-grade cervical dysplasia screening and management.

Risk-based management of cervical dysplasia emerged as an effective approach for individualized management of patients that allows specific individual characteristics such as age and patients’ concerns about the potential impact of treatment on pregnancy outcomes to guide the specific therapeutic approach, while maintaining a low risk for adverse outcomes. However, specific evolution of cervical dysplasia is often challenging and difficult to predict, and numerous additional tools are currently under investigation for improving the patients’ risk stratification and prognosis.

Biomolecular tests such as p16/Ki67 dual staining and methylation markers to specific artificial intelligence-based methods have shown promising results for improving the diagnosis and prediction of cervical dysplasia progression, persistence, or regression [10,11,12,13]. However, these approaches are limited by high costs and accessibility constraints.

A competing risks approach quantifies the cumulative incidence of each distinct occurrence, accurately considering the existence of alternative outcomes. Fine and Gray’s subdistribution hazard model facilitates the calculation of covariate effects regarding the likelihood of encountering each event type over time, while accounting for other occurrences as competing hazards [14]. This approach produces more clinically significant and interpretable risk estimates, facilitating the categorization of patients based on overall risk as well as the specific probabilities of progression, reversal, or persistence. This modeling can lead to more nuanced management strategies, directing decisions on surveillance versus rapid therapy and identifying subgroups at elevated risk of adverse outcomes.

The aim of this study was to test the prognostic utility of immediate pre-treatment and post-treatment risk predictions, derived from the American Society of Colposcopy and Cervical Pathology (ASCCP) risk-based management guidelines for the prediction of progression, regression, or persistence of high-grade cervical dysplasia in a cohort of patients from Romania.

## 2. Materials and Methods

We conducted a prospective cohort study of women aged over 30 years who underwent self-referred or targeted screening with or without histologically confirmed CIN of any grade. Participants were recruited from colposcopy clinics across the Moldavian region, Romania, between 2021 and 2024. Approval for this study was obtained from the Institutional Ethics Committee of Clinical Hospital of Obstetrics and Gynecology, “Buna vestire” Galati (No. 115/5 January 2021). All participants provided written informed consent.

Inclusion criteria included age > 30 years and at least one baseline evaluation with cervical cytology, HPV genotyping, and/or histopathology. The participants agreed to co-testing and offered their informed consent to provide their medical data for further analysis in this study. The exclusion criteria included prior cervical cancer, hysterectomy, and needing specific oncological and surgical treatment for confirmed cervical cancer (these patients were referred to the regional institutes of oncology from Romania).

At enrolment, patients either provided the results of Pap testing and/or HPV genotyping, undergoing specific cervical screening (the Pap test and HPV genotyping were processed with the ThinPrep liquid-based method (ThinPrep-Hologic, Bedford, MA, USA). The results from cytology testing was classified as Negative for Intraepithelial Lesion or Malignancy (NILM); Atypical Squamous Cells of Undetermined Significance (ASC-US), Atypical Squamous Cells That Cannot Exclude High-Grade Squamous Intraepithelial Lesion (ASC-H); Low-Grade Squamous Intraepithelial Lesion (LSIL); High-Grade Squamous Intraepithelial Lesion (HSIL); and Squamous Cell Carcinoma (SCC).

Patients diagnosed with HPV-negative NILM served as the control group and were included in further routine follow-up for 5 years; patients with a positive HPV test result and NILM, ASC-US, or LSIL underwent repeated HPV (1 year) and cytology testing (at 6 months) and were referred for colposcopy in case of persistence or progression of cervical dysplasia; while HPV-positive patients with a cervical cytology of HSIL or worse underwent colposcopy, which was performed by certified physicians from Galati, Iasi, or Bucharest, and punch biopsy (PB), cone biopsy, or Large-Loop Excision of the Transformation Zone (LLETZ). The indications for cervical biopsy in patients with high-risk HPV included the following: (1) abnormal Pap smear results (e.g., ASC-US, LSIL, HSIL) to confirm and grade the presence of CIN; (2) suspicious colposcopic findings; (3) to determine the need for excisional procedures. The indications for LLETZ in patients with high-risk HPV included the following: (1) HSIL/CIN2-3 diagnosis; (2) discrepancy between cytology and histology; (3) unsatisfactory colposcopy.

The follow-up for the purpose of this study was 24 months and included repeated cytology testing, HPV genotyping and, in specific cases, with repeated colposcopy, biopsy and/or excisional treatment according to the national protocol. Follow-up continued until an outcome of interest occurred or censoring was implemented due to loss to follow-up or the study coming to an end. We used the ASCCP Management Guidelines Web Application (available at: https://app.asccp.org/ (accessed on 13 March 2025).) to determine the immediate risk of CIN3+ at routine screening and post-treatment for patients. A cut-off of 60% was further used in the competing risk approach for further patient stratification.

The clinical data of the patients included in this study comprised age, smoking habits, use of oral contraception, parity, immunosuppression, the presence of arterial hypertension and diabetes, and personal history of sexually transmitted infections.

Progression is defined as histologically confirmed worsening of the CIN grade or a diagnosis of invasive cervical cancer. Also, regression of lesions was defined as a histological diagnostic of CIN1/ CIN2 that was modified to negative/CIN1 at follow-up. The same histopathological finding during follow-up showed lesion persistence [11]. Participants who did not have any of these outcomes by the last follow-up were considered to be censored.

We summarized baseline characteristics using means (±standard deviations—SDs) or medians (interquartile ranges—IQRs) for continuous variables and frequencies (percentages) for categorical variables. We compared mean immediate pre-treatment and post-treatment risk scores across outcome categories using one-way analysis of variance (ANOVA). Post hoc pairwise comparisons were performed using Bonferroni correction to adjust for multiple testing.

To analyze the time to each outcome while accounting for competing risks, we employed Fine and Gray’s subdistribution hazard model. This approach models the cumulative incidence function for each specific outcome (progression, regression, and persistence), treating other outcomes as competing events. We specified the time from baseline evaluation to the first occurrence of progression, regression, or persistence as the primary time-to-event outcome. Immediate pre-treatment and post-treatment predicted risk scores were included as continuous predictors. Censoring occurred at last follow-up for patients without any event or who were lost to follow-up. These outcomes were stratified depending on the type of high-grade CIN: CIN2+ and CIN3+.

To evaluate the impact of risk thresholds, we also dichotomized scores at >60% and analyzed these categorical predictors. Results were presented as SHRs (subdistribution hazard ratios) with 95% confidence intervals (CIs).

We estimated and plotted cumulative incidence functions stratified by immediate pre- and post-treatment risk > 60% and by histology subgroup (CIN2+ and CIN3+) for each outcome to illustrate predicted outcome probabilities over time while accounting for competing risks.

Analyses were conducted using Stata version 19.5 (StataCorp, College Station, TX, USA). A two-sided *p*-value < 0.05 was considered statistically significant.

## 3. Results

The baseline characteristics of the study population, stratified by lesion outcome at follow-up (censored, progression, regression, and persistence), are presented in Table 1. The final cohort for analysis included 223 patients, comprising 124 with censored outcomes, 46 patients with progression of cervical dysplasia, 25 patients with regression of cervical dysplasia, and 28 patients with persistent lesions. The mean age of participants was similar across groups (overall mean 44.3 ± 10.4 years, *p* = 0.402), with no significant differences observed between groups. Parity also did not differ significantly among groups (overall mean 2.09 ± 1.91 births, *p* = 0.545), and the same findings were applied to personal history of hypertension (*p* = 0.393) and diabetes (*p* = 0.911).

Smoking habit was more frequently encountered among patients with progression (32.6%), regression (44.0%), and persistence (32.1%) than among those who were censored (17.7%, *p* = 0.016). Also, the use of combined oral contraceptives (COCs) was more frequent in the progression group (21.7%) compared with the other groups (*p* = 0.019).

Immunosuppression was also significantly associated with lesion outcome (*p* = 0.021), being present in 15.2% of women with progression, in 8% of those with regression and in 10.7% of those with persistence compared to only 2.4% in the censored group. The history of sexually transmitted infections was not statistically different between groups (*p* = 0.095).

**Figure 1:**
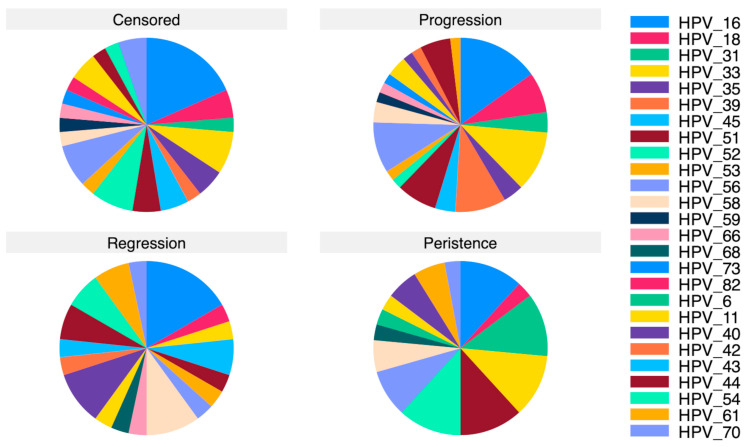
Schematic representation of HPV strain distribution among groups.

Figure 1 represents the distribution of HPV strains among the evaluated groups. HPV16 was significantly more prevalent among patients with persistent lesions (14.3%), progression (17.4%), and regression (20.0%) than among censored patients (5.6%, *p* = 0.045). Also, HPV31 and HPV33 strains demonstrated significantly higher frequencies among patients with persistence (HPV31—14.3%, HPV33—14.3%) or progression (HPV31—4.4%, HPV33—13%) of high-grade cervical dysplasia compared with censored outcomes (HPV31—0.8%, *p* = 0.002, HPV33—2.4%, *p* = 0.019). See Supplementary Table S1, which illustrates the distribution of HPV genotypes by lesion outcome.

Other HPV strains that were significantly more prevalent in the progression and/or persistence groups included HPV51 (*p* = 0.023), HPV52 (*p* = 0.012), HPV58 (*p* = 0.029), and HPV61 (*p* = 0.021).

Table 2 comparatively outlines the distribution of HPV strains among the evaluated groups. The high-risk and probable high-risk HPV strains (HPV16, 18, 26, 31, 33, 35, 39, 45, 51, 52, 53, 56, 58, 59, 66, 68, 69, 73 and 82) were significantly more prevalent among patients who experienced the progression (65.2%) and persistence (71.4%) of high-risk cervical dysplasia (*p* < 0.001). On the other hand, the highest prevalence of low-risk HPV strains (HPV6, 11, 40, 42, 43, 44, 54, 61 and 70) was encountered in the group that experienced regression (48%, *p* < 0.001). At the same time, multiple infections were significantly more frequently encountered in patients with progression (32.6%) and persistence (28.6%) of cervical dysplasia (*p* = 0.002).

In Table 3, we present the distribution of baseline histologic and cytologic categories across follow-up outcomes. Our data indicate that CIN1 was more frequent in regression (32%) than in any other group (censored 1.6%, persistence 3.5%, progression 0%). CIN2 was significantly higher in the persistence (53.5%) and regression (36%) groups. On the other hand, CIN3+ (36.9%) was significantly more frequently encountered in the progression group, while the highest rates of CIN2+ were encountered in the persistence group.

Regarding the cytological categories, HSIL cytology was significantly more common among progression cases (36.9%) compared to censored cases (2.4%). LSIL cytology was more frequent in regression cases (40.0%).

**Figure 2:**
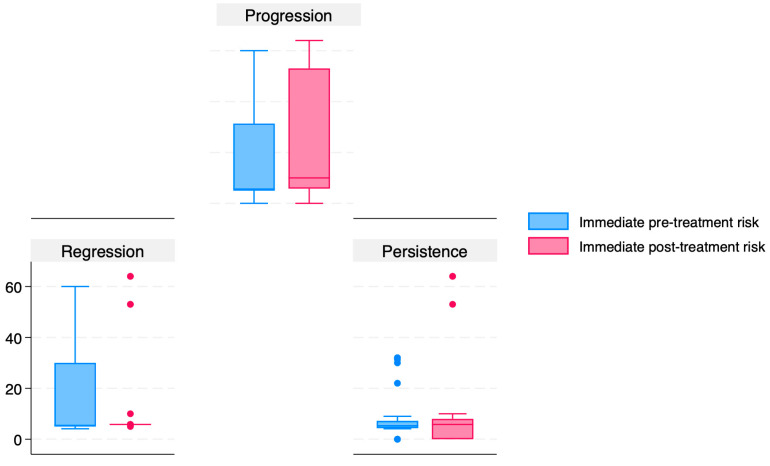
Boxplots representing the immediate pre-treatment and post-treatment calculated risk stratified by outcome.

In Table 4, we present the results of the ANOVA analyses that compared the immediate pre-treatment and post-treatment calculated risks. A graphical representation of this comparison is shown in Figure 2. Our data indicate that the mean immediate pre-treatment and post-treatment risks were significantly higher for those patients who had high-grade cervical dysplasia regression and progression (*p* < 0.001) compared to those who experienced persistence.

In Table 5, we presented the results from the post hoc pairwise comparisons using Bonferroni correction for the immediate pre-treatment and post-treatment calculated risks. Our data indicated that the immediate pre-treatment calculated risk was significantly higher in the group that later experienced progression compared to the group that experienced persistence (risk difference: −8.25, *p* = 0.042). Moreover, the immediate post-treatment calculated risk was significantly higher for those with progression of cervical dysplasia compared to those who experienced regression (risk difference: −13.93, *p* = 0.004) and persistence (risk difference: −18.01, *p* < 0.001).

In Table 6, we presented the SHR from competing-risks regressions models that used the immediate pre-treatment and post-treatment calculated risks as predictors. Our data indicated that immediate post-treatment risk was significantly associated with progression (SHR = 1.022, 95% CI = 1.008–1.036, *p* = 0.002), suggesting it is a strong predictor of disease progression.

Immediate pre-treatment risk was significantly associated with regression (SHR = 1.022, 95% CI = 1.002–1.043, *p* = 0.031), indicating higher-baseline risk may predict subsequent regression following treatment. Neither pre- nor post-treatment risk scores were significant predictors of persistence of high-grade cervical dysplasia.

When we used a >60% cut-off value for the evaluated risk, we found out that both immediate pre-treatment (SHR = 4.13, 95% CI = 1.15–14.87, *p* = 0.030) and post-treatment (SHR = 2.55, 95% CI = 1.06–6.11, *p* = 0.036) risks were significantly associated with increased risk of progression. However, neither predictor was significantly associated with regression (*p* > 0.5). For persistence, the immediate pre-treatment risk was a significant predictor (SHR = 3.90, 95% CI = 1.34–6.14, *p* < 0.001).

In Table 7, we present the SHRs from competing-risks regressions stratified by the type of high-grade dysplasia considering immediate pre-treatment and post-treatment calculated risks with a cut-off value of >60% as predictors.

In the CIN3+ subgroup, an immediate pre-treatment predicted risk greater than 60% was associated with an almost eightfold increase in the hazard of progression (SHR 7.97; 95% CI, 4.09–19.03; *p* < 0.001). Immediate post-treatment predicted risk above 60% also remained a significant predictor of progression, with a doubling of the hazard (SHR 2.08; 95% CI, 1.52–2.85; *p* < 0.001). Among patients with CIN2+, post-treatment risk > 60% was similarly associated with a substantially increased progression risk (SHR 3.47; 95% CI, 1.23–9.83; *p* = 0.019).

Predicted risk scores greater than 60% did not significantly predict regression in any histologic subgroup. These scores, however, were strong predictors of disease persistence across histologic subgroups. Among patients with CIN2+, both pre-treatment and post-treatment predicted risk greater than 60% were associated with approximately fivefold increases in the hazard of persistence (pre-treatment SHR 5.19; 95% CI, 1.55–17.4; *p* < 0.001; post-treatment SHR 5.15; 95% CI, 1.95–13.31; *p* < 0.001). Similar associations were observed in the CIN3+ subgroup (*p* < 0.001).

**Figure 3:**
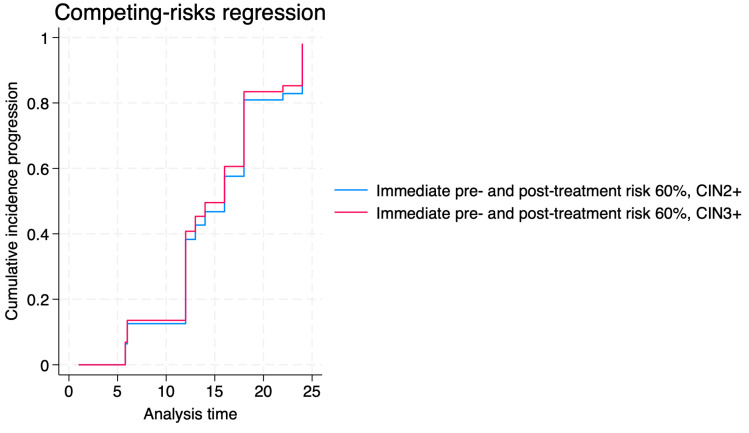
Cumulative incidence of progression of CIN2+ and CIN3+ for immediate pre- and post-treatment risks > 60%.

**Figure 4:**
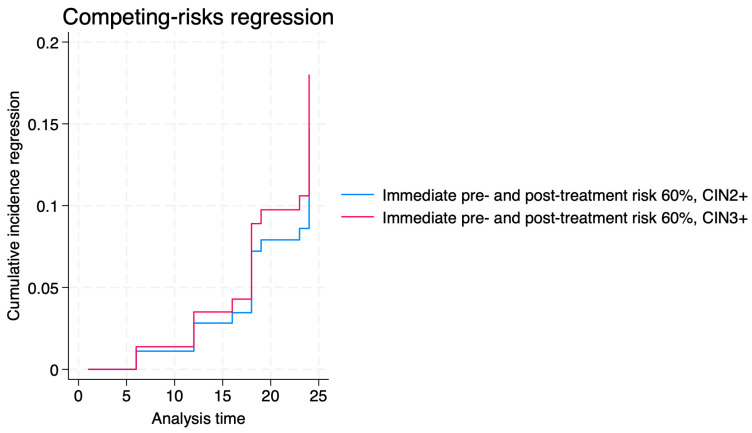
Cumulative incidence of regression of CIN2+ and CIN3+ for immediate pre- and post-treatment risks > 60%.

**Figure 5:**
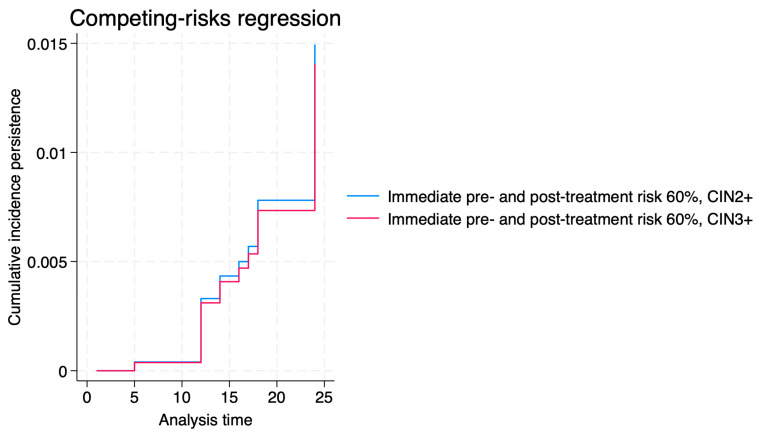
Cumulative incidence of persistence of CIN2+ and CIN3+ for immediate pre- and post-treatment risks > 60%.

Figure 3, Figure 4 and Figure 5 show the cumulative incidence of progression, regression, and persistence of CIN2+ and CIN3+ for immediate pre- and post-treatment risks > 60%. Our data revealed that the progression risk was high in both groups, with minimal separation between curves. The regression risk curves showed slightly higher cumulative incidence for CIN3+ than CIN2+, though differences were not statistically significant. Persistence risk remained low overall for the established threshold, without significant differences noted between strata.

## 4. Discussion

In this prospective cohort study, we evaluated the prognostic utility of immediate pre-treatment and post-treatment risk estimates derived from the ASCCP risk-based management guidelines [15] for the prediction of progression, regression, or persistence of high-grade cervical dysplasia.

Our data showed that immediate post-treatment risk was significantly associated with subsequent progression of cervical dysplasia, and this observation was consistent across continuous and dichotomous (>60%) risk thresholds. For a cut-off of 60%, the immediate post-treatment risk was able to significantly predict the progression of both CIN2+ and CIN3+.

On the other hand, an immediate pre-treatment risk of > 60% was significantly associated with progression of CIN3+, but not of CIN2+. Also, the immediate pre-treatment risk was significantly associated with regression, but this observation did not persist at the >60% threshold.

While continuous risk scores did not accurately predict the overall persistence of high-grade cervical dysplasia, when a >60% cut-off was applied, it improved the discriminative power. Thus, both pre- and post-treatment risk > 60% were strongly associated with persistence across histologic subgroups.

As far as we know, this is the first prospective study that evaluated the prognostic performance of the ASCCP risk scores in an Eastern European population. Comparative data from non-U.S. cohorts are scarce [16], particularly those examining longitudinal outcomes beyond initial risk triage (progression, regression, and persistence).

These risk scores and risk management strategies have been validated in large cohorts such as the Kaiser Permanente Northern California (KPNC) cohort (approximately 1.5 million participants), the New Mexico HPV Pap Registry (450,000 participants) CDC NBCCEDP (approximately 200,000 participants), and the BD Onclarity Trial (approximately 30,000 participants) [17,18].

A retrospective study by Zhao et al. that included 96,318 patients aged 25–65 years who underwent co-testing aimed to evaluate the CIN3+ risk and the portability of ASCCP risk-based management [19]. The authors showed that the CIN3+ immediate risk varied between subgroups of positive HPV16 (34.09%), HPV18 (13.38%), other HPV types (6.71%), and negative hrHPV (0.12%). Also, the authors compared their results to those determined in the KPNC cohort, showing a significantly higher CIN3+ immediate risk (1.42% vs. 0.46%) and disproportionately increased cancer immediate risks in most subgroups requiring immediate colposcopy or treatment [19].

The immediate and cumulative CIN3+ risk fluctuates depending on numerous variables that could include screening participants, screening methodologies, and the management of screening outcomes. In regions with elevated cervical cancer prevalence, such as Romania and Eastern Europe, screening tends to be more opportunistic than systematic, and those undergoing opportunistic screening typically exhibit greater (pre)cancer risks than the overall population [20,21].

We also found significant associations between certain baseline clinical characteristics and lesion outcomes. Thus, smoking, immunosuppression, and use of combined oral contraceptives were more common in patients with progression and persistence, aligning with prior literature on behavioral and hormonal cofactors in cervical carcinogenesis. Additionally, high-risk HPV genotypes such as HPV16, 31, 33, and 39, were significantly more prevalent among patients with progressive or persistent lesions [22,23]. Conversely, low-risk HPV types were more frequent in the regression group. The presence of multiple HPV strains was also significantly associated with adverse outcomes, indicating that co-infections may potentiate oncogenic transformation or impair clearance [24]. Moreover, age appears to be a clinical characteristic with significant impact on the lesions’ progression or regression [25].

Our study outlined the applicability and limitations of ASCCP risk tools in an Eastern European setting. In Romania, where cervical cancer incidence remains among the highest in the European Union, a risk-based management strategy could support more precise allocation of limited resources and reduce overtreatment. Moreover, we would like to point out that none of the participants were vaccinated. On the other hand, our findings also highlight the need to adapt these models for local populations, considering differences in HPV genotype distribution, access to colposcopy, and population immunity (including vaccination status). Limitations of this study include the small cohort size, loss to follow-up, and lack of validation of these risk scores in the evaluated population.

Future research could include multiple predictors in models that evaluate the prognostic utility of these risk scores. For example, Xue and colleagues conducted a multicenter, cross-sectional study of a Chinese population that analyzed medical data and risk scores based on ASCCP guidelines from 6012 patients who underwent cervical cancer screening and colposcopic evaluation [26]. Their data indicated that the risk of CIN3+ ranged from 0% for normal/benign colposcopic impressions, <HSIL cytologies, and HPV negative to 63.61% for high-grade colposcopy, HSIL+ cytology, and HPV16/18+, across 18 subgroups. Moreover, they indicated that high-grade colposcopic impressions were associated with a >19% increased risk of CIN3+, even in participants without HSIL+ cytology and/or HPV16/18+ [26].

## 5. Conclusions

In the evaluated cohort, the ASCCP-derived immediate risk estimates, especially post-treatment risk > 60%, proved effective in predicting progression and persistence of high-grade cervical dysplasia.

These findings support the use of risk-based tools for individualized follow-up and management, even in opportunistic screening settings.

However, local validation and model adaptation are needed to account for regional differences in HPV epidemiology and healthcare access.

## Figures and Tables

**Table 1:** Baseline characteristics of the study population.

Variable	Censored (N = 124)	Progression (N = 46)	Regression (N = 25)	Persistence (N = 28)	Total (N = 223)	*p*-Value
Age, mean (SD)	44.9 (10.7)	44.1 (10.6)	45.1 (10.5)	41.3 (9.0)	44.3 (10.4)	0.402
Parity, mean (SD)	2.02 (1.88)	2.04 (1.98)	1.92 (1.53)	2.57 (2.27)	2.09 (1.91)	0.545
HTN (N, %)	6 (4.8%)	4 (8.7%)	2 (8.0%)	0 (0.0%)	12 (5.4%)	0.393
Diabetes (N, %)	5 (4.0%)	2 (4.4%)	1 (4.0%)	2 (7.1%)	10 (4.5%)	0.911
Smoker (N, %)	22 (17.7%)	15 (32.6%)	11 (44.0%)	9 (32.1%)	57 (25.6%)	0.016 *
COC use (N, %)	7 (5.6%)	10 (21.7%)	2 (8.0%)	3 (10.7%)	22 (9.9%)	0.019 *
Immunosuppression (N, %)	3 (2.4%)	7 (15.2%)	2 (8.0%)	3 (10.7%)	15 (6.7%)	0.021 *
History of STI (N, %)	4 (3.2%)	6 (13.0%)	2 (8.0%)	1 (3.6%)	13 (5.8%)	0.095

Legend: SD—standard deviation; HTN—chronic hypertension; COC—combined oral contraceptive; STI—sexually transmitted infections. Asterisks indicate statistically significant differences (*p* < 0.05).

**Table 2:** Comparative analysis of HPV strains distribution among groups.

Variable	Progression (N = 46)	Regression (N = 25)	Persistence (N = 28)	Censored (N = 124)	*p*-Value
HPV high risk (n/%)	30 (65.2%)	13 (52.0%)	20 (71.4%)	84 (37.7%)	<0.001
HPV low risk (n/%)	8 (17.4%)	12 (48.0%)	7 (25.0%)	33 (14.8%)	<0.001
Multiple HPV strains (n/%)	15 (32.6%)	3 (12.0%)	8 (28.6%)	39 (17.5%)	0.002

Legend: HPV—human papillomavirus.

**Table 3:** The distribution of baseline histologic and cytologic categories among groups.

Test	Category	Censored (N = 124)	Progression (N = 46)	Regression (N = 25)	Persistence (N = 28)	*p*-Value
Histology	CIN1	2 (1.61)	0 (0.0)	8 (32)	1 (3.5)	<0.001
	CIN2	6 (4.8)	2 (4.3)	9 (36)	15 (53.5)	
	CIN2+	10 (8.06)	19 (41.3)	11 (44)	19 (67.8)	
	CIN3	3 (2.4)	16 (34.78)	2 (8)	4 (14.2)	
	CIN3+	4 (3.2)	17 (36.9)	2 (8)	4 (14.2)	
Cytology	NILM	103 (83)	13 (28.2)	2 (8.0)	9 (32.1)	<0.001
	ASC-US	8 (6.4)	5 (10.8)	4 (16.0)	7 (25.0)	
	LSIL	7 (5.6)	7 (15.2)	10 (40.0)	6 (21.4)	
	HSIL	3 (2.4)	17 (36.9)	7 (28.0)	5 (17.9)	
	ASC-H	2 (1.6)	3 (6.5)	2 (8.0)	1 (3.6)	
	SCC	1 (0.8)	1 (2.1)	0 (0.0)	0 (0.0)	

Legend: CIN1 = Cervical Intraepithelial Neoplasia grade 1; CIN2 = Cervical Intraepithelial Neoplasia grade 2; CIN2+ = Cervical Intraepithelial Neoplasia grade 2 or worse; CIN3+ = Cervical Intraepithelial Neoplasia grade 3 or carcinoma in situ; NILM = Negative for Intraepithelial Lesion or Malignancy; ASC-US = Atypical Squamous Cells of Undetermined Significance; LSIL = Low-Grade Squamous Intraepithelial Lesion; HSIL = High-Grade Squamous Intraepithelial Lesion; ASC-H = Atypical Squamous Cells, cannot exclude HSIL; SCC—squamous cell carcinoma.

**Table 4:** Description of immediate pre-treatment and post-treatment calculated risks stratified by outcome (ANOVA analysis).

Status Category	Risk Mean (SD)	*p*-Value	Post-Treatment Risk Mean (SD)	*p*-Value
Progression	18.14 (16.86)	<0.001	28.30 (25.67)	<0.001
Regression	15.07 (14.96)		14.37 (19.80)	
Persistence	9.90 (10.92)		10.29 (18.07)	

Legend: SD—standard deviation.

**Table 5:** Post hoc pairwise comparisons using Bonferroni correction for the immediate pre-treatment and post-treatment calculated risks.

Comparison	Immediate Pre-Treatment Risk Difference	*p*-Value	Immediate Post-Treatment Risk Difference	*p*-Value
Progression vs. Regression	−3.08	1.000	−13.93	0.004
Progression vs. Persistence	−8.25	0.042	−18.01	<0.001
Regression vs. Persistence	−5.17	0.832	−4.08	1.000

**Table 6:** Subdistribution hazard ratios from competing-risks regressions considering immediate pre-treatment and post-treatment calculated risks as predictors.

Failure Type	Predictor	SHR (95% CI)	*p*-Value	Predictor	SHR (95% CI)	*p*-Value
Progression	Immediate pre-treatment risk	1.015 (0.993–1.038)	0.181	Immediate pre-treatment risk > 60%	4.13 (1.15–14.87)	0.030
	Immediate post-treatment risk	1.022 (1.008–1.036)	0.002	Immediate post-treatment risk > 60%	2.55 (1.06–6.11)	0.036
Regression	Immediate pre-treatment risk	1.022 (1.002–1.043)	0.031	Immediate pre-treatment risk > 60%	1.55 (0.30–7.95)	0.601
	Immediate post-treatment risk	0.994 (0.974–1.013)	0.520	Immediate post-treatment risk > 60%	1.49 (0.32–6.93)	0.609
Persistence	Immediate pre-treatment risk	1.005 (0.982–1.029)	0.673	Immediate pre-treatment risk > 60%	3.90 (1.34–6.14)	<0.001
	Immediate post-treatment risk	0.991 (0.969–1.013)	0.427	Immediate post-treatment risk > 60%	1.24 (0.33–4.70)	0.751

Legend: SHR = subdistribution hazard ratio from Fine and Gray competing-risks regression, CI = confidence interval.

**Table 7:** Subdistribution hazard ratios from competing-risks regressions stratified by the type of high-grade dysplasia considering immediate pre-treatment and post-treatment calculated risks with a cut-off value > 60% as predictors.

Outcome	Predictor	Histology Subgroup	SHR (95% CI)	*p*-Value
Progression	Immediate pre-treatment risk > 60%	CIN2+	2.05 (0.51–8.24)	0.312
	Immediate pre-treatment risk > 60%	CIN3+	7.97 (4.09–19.03)	<0.001
	Immediate post-treatment risk > 60%	CIN2+	3.47 (1.23–9.83)	0.019
	Immediate post-treatment risk > 60%	CIN3+	2.08 (1.52–2.85)	<0.001
Regression	Immediate pre-treatment risk > 60%	CIN2+	1.22 (0.24–6.25)	0.813
	Immediate pre-treatment risk > 60%	CIN3+	1.01 (0.97–7.56)	0.774
	Immediate post-treatment risk > 60%	CIN2+	0.81 (0.11–6.03)	0.841
	Immediate post-treatment risk > 60%	CIN3+	0.73 (0.16–18.20)	0.649
Persistence	Immediate pre-treatment risk > 60%	CIN2+	5.19 (1.55– 17.4)	<0.001
	Immediate pre-treatment risk > 60%	CIN3+	5.23 (1.99– 19.16)	<0.001
	Immediate post-treatment risk > 60%	CIN2+	5.15 (1.95– 13.31)	<0.001
	Immediate post-treatment risk > 60%	CIN3+	5.19 (1.97– 13.87)	<0.001

Legend: SHR = subdistribution hazard ratio from Fine and Gray competing-risks regression; CI = confidence interval; CIN2+ = Cervical Intraepithelial Neoplasia grade 2 or worse; CIN3+ = Cervical Intraepithelial Neoplasia grade 3 or carcinoma in situ.

## Data Availability

The datasets are available from the correspondent authors upon reasonable request due to local policies.

## Supplementary Materials

The following supporting information can be downloaded at https://www.mdpi.com/article/10.3390/jcm14176303/s1, Table S1: Distribution of HPV genotypes by lesion outcome. Table S2: Distribution of high-risk HPV genotypes by histology of cervical lesions.

## Abbreviations

The following abbreviations are used in this manuscript:

AI	Artificial Intelligence.
ANOVA	Analysis of Variance.
ASC-H	Atypical Squamous Cells That Cannot Exclude High-Grade Squamous Intraepithelial Lesion.
ASC-US	Atypical Squamous Cells of Undetermined Significance.
ASCCP	American Society for Colposcopy and Cervical Pathology.
CDC	Centers for Disease Control and Prevention.
CI	Confidence Interval.
CIN	Cervical Intraepithelial Neoplasia.
CIN2+	Cervical Intraepithelial Neoplasia grade 2 or worse.
CIN3+	Cervical Intraepithelial Neoplasia grade 3 or carcinoma in situ.
CIS	Carcinoma in Situ.
COC	Combined Oral Contraceptives.
EU/EEA	European Union / European Economic Area.
HGSIL	High-Grade Squamous Intraepithelial Lesion.
HPV	Human Papillomavirus.
hrHPV	High-Risk Human Papillomavirus.
HSIL	High-Grade Squamous Intraepithelial Lesion.
HTN	Hypertension.
KPNC	Kaiser Permanente Northern California.
LGSIL	Low-Grade Squamous Intraepithelial Lesion.
LLETZ	Large Loop Excision of the Transformation Zone.
LSIL	Low-Grade Squamous Intraepithelial Lesion.
NBCCEDP	National Breast and Cervical Cancer Early Detection Program.
NILM	Negative for Intraepithelial Lesion or Malignancy.
PB	Punch Biopsy.
p16/Ki67	(Dual immunohistochemical) p16 and Ki-67 proteins.
SCC	Squamous Cell Carcinoma.
SD	Standard Deviation.
SHR	Subdistribution Hazard Ratio.
STI	Sexually Transmitted Infection.
WHO	World Health Organization.
