# Supplementary material for: A Competing-Risks Approach to the Progression, Regression and Persistence of High-Grade Cervical Dysplasia in Patients over 30 Years Old—A Prospective Study
Source: J Clin Med. 2025 Sep 6;14(17):6303. doi: 10.3390/jcm14176303 (PMC12429521; doi:10.3390/jcm14176303)
Supplement: Supplementary file 1 [file jcm-14-06303-s001.zip › jcm-3794467-supplementary.pdf]

Supplementary table

Table S1. Distribution of HPV genotypes by lesion outcome

HPV strain	Censored N (%)	Progression N (%)	Regression N (%)	Persistence N (%)	Total N (%)	p-value
HPV_11	2 (1.6)	2 (4.4)	1 (4.0)	1 (3.6)	6 (2.7)	0.734
HPV_6	0 (0.0)	0 (0.0)	0 (0.0)	1 (3.6)	1 (0.4)	0.072
HPV_16	7 (5.6)	8 (17.4)	5 (20.0)	4 (14.3)	24 (10.8)	0.045*
HPV_18	2 (1.6)	4 (8.7)	1 (4.0)	1 (3.6)	8 (3.6)	0.181
HPV_31	1 (0.8)	2 (4.4)	0 (0.0)	4 (14.3)	7 (3.1)	0.002*
HPV_33	3 (2.4)	6 (13.0)	1 (4.0)	4 (14.3)	14 (6.3)	0.019*
HPV_34	1 (0.8)	0 (0.0)	0 (0.0)	0 (0.0)	1 (0.4)	0.849
HPV_35	2 (1.6)	2 (4.4)	0 (0.0)	0 (0.0)	4 (1.8)	0.441
HPV_39	1 (0.8)	5 (10.9)	0 (0.0)	0 (0.0)	6 (2.7)	0.002*
HPV_40	0 (0.0)	1 (2.2)	3 (12.0)	2 (7.1)	6 (2.7)	0.003*
HPV_42	0 (0.0)	1 (2.2)	1 (4.0)	0 (0.0)	2 (0.9)	0.177
HPV_43	0 (0.0)	0 (0.0)	1 (4.0)	0 (0.0)	1 (0.4)	0.047*
HPV_44	1 (0.8)	3 (6.5)	2 (8.0)	0 (0.0)	6 (2.7)	0.052
HPV_45	2 (1.6)	2 (4.4)	2 (8.0)	0 (0.0)	6 (2.7)	0.212
HPV_51	2 (1.6)	4 (8.7)	1 (4.0)	4 (14.3)	11 (4.9)	0.023*
HPV_52	3 (2.4)	1 (2.2)	0 (0.0)	4 (14.3)	8 (3.6)	0.012*
HPV_53	1 (0.8)	1 (2.2)	1 (4.0)	0 (0.0)	3 (1.3)	0.528
HPV_54	1 (0.8)	0 (0.0)	2 (8.0)	0 (0.0)	3 (1.3)	0.022*
HPV_56	3 (2.4)	5 (10.9)	1 (4.0)	3 (10.7)	12 (5.4)	0.089
HPV_58	1 (0.8)	2 (4.4)	3 (12.0)	2 (7.1)	8 (3.6)	0.029*
HPV_59	1 (0.8)	1 (2.2)	0 (0.0)	0 (0.0)	2 (0.9)	0.721
HPV_61	0 (0.0)	1 (2.2)	2 (8.0)	2 (7.1)	5 (2.2)	0.021*
HPV_65	0 (0.0)	1 (2.2)	0 (0.0)	0 (0.0)	1 (0.4)	0.276
HPV_66	1 (0.8)	1 (2.2)	1 (4.0)	0 (0.0)	3 (1.3)	0.528
HPV_68	0 (0.0)	0 (0.0)	1 (4.0)	1 (3.6)	2 (0.9)	0.090
HPV_70	2 (1.6)	0 (0.0)	1 (4.0)	1 (3.6)	4 (1.8)	0.561
