# Supplementary material for: A Competing-Risks Approach to the Progression, Regression and Persistence of High-Grade Cervical Dysplasia in Patients over 30 Years Old—A Prospective Study
Source: J Clin Med. 2025 Sep 6;14(17):6303. doi: 10.3390/jcm14176303 (PMC12429521; doi:10.3390/jcm14176303)
Supplement: Supplementary file 1 [file jcm-14-06303-s001.zip › S2.pdf]

Supplementary table

Table S2. Distribution of high-risk HPV genotypes by histology of cervical lesions

HPV strain	CIN1 (11 patients)	CIN2 (32 patients)	CIN3 (25 patients)
HPV_16	-	5 (15.6)	8 (32)
HPV_18	1 (9.09)	3 (9.36)	2 (8)
HPV_31	1 (9.09)	8 (25)	11 (44)
HPV_33	2 (18.18)	5 (15.6)	1 (4)
HPV_35	-	2 (6.25)	-
HPV_39	1 (9.09)	-	-
HPV_45	-	2 (6.25)	-
HPV_51	2 (18.18)	4 (12.48)	1 (4)
HPV_52	-	3 (9.36)	1 (4)
HPV_56	-	-	
HPV_58	-	-	1 (4)
HPV_59	1 (9.09)	1 (3.12)	-
HPV_68	-	-	-
